# Mitochondrial Enzymes of the Urea Cycle Cluster at the Inner Mitochondrial Membrane

**DOI:** 10.3389/fphys.2020.542950

**Published:** 2021-01-21

**Authors:** Nantaporn Haskins, Shivaprasad Bhuvanendran, Claudio Anselmi, Anna Gams, Tomas Kanholm, Kristen M. Kocher, Jonathan LoTempio, Kylie I. Krohmaly, Danielle Sohai, Nathaniel Stearrett, Erin Bonner, Mendel Tuchman, Hiroki Morizono, Jyoti K. Jaiswal, Ljubica Caldovic

**Affiliations:** ^1^Center for Genetic Medicine Research, Children’s National Medical Center, Washington, DC, United States; ^2^Department of Genomics and Precision Medicine, School of Medicine and Health Sciences, The George Washington University, Washington, DC, United States; ^3^Department of Biomedical Engineering, School of Engineering and Applied Sciences, The George Washington University, Washington, DC, United States; ^4^School of Medicine and Health Sciences, Institute for Biomedical Sciences, The George Washington University, Washington, DC, United States; ^5^Computational Biology Institute, Milken Institute School of Public Health, The George Washington University, Washington, DC, United States

**Keywords:** urea cycle, N-acetylglutamate synthase, carbamylphosphate synthetase 1, ornithine transcarbamylase, enzyme cluster, mitochondria, metabolite channeling, super-resolution imaging

## Abstract

Mitochondrial enzymes involved in energy transformation are organized into multiprotein complexes that channel the reaction intermediates for efficient ATP production. Three of the mammalian urea cycle enzymes: N-acetylglutamate synthase (NAGS), carbamylphosphate synthetase 1 (CPS1), and ornithine transcarbamylase (OTC) reside in the mitochondria. Urea cycle is required to convert ammonia into urea and protect the brain from ammonia toxicity. Urea cycle intermediates are tightly channeled in and out of mitochondria, indicating that efficient activity of these enzymes relies upon their coordinated interaction with each other, perhaps in a cluster. This view is supported by mutations in surface residues of the urea cycle proteins that impair ureagenesis in the patients, but do not affect protein stability or catalytic activity. We find the NAGS, CPS1, and OTC proteins in liver mitochondria can associate with the inner mitochondrial membrane (IMM) and can be co-immunoprecipitated. Our *in-silico* analysis of vertebrate NAGS proteins, the least abundant of the urea cycle enzymes, identified a protein-protein interaction region present only in the mammalian NAGS protein—“variable segment,” which mediates the interaction of NAGS with CPS1. Use of super resolution microscopy showed that NAGS, CPS1 and OTC are organized into clusters in the hepatocyte mitochondria. These results indicate that mitochondrial urea cycle proteins cluster, instead of functioning either independently or in a rigid multienzyme complex.

## Introduction

Mitochondria are ATP producing organelles where multi-enzyme complexes catalyze reactions of the TCA cycle, fatty acid beta-oxidation and oxidative phosphorylation. To perform these diverse functions, mitochondria maintain the inner and outer mitochondrial membrane-bound compartments. These compartments are further spatially sub-organized into complexes to efficiently channel unstable and highly reactive intermediates between enzymes that catalyze consecutive reactions of the TCA cycle, oxidative phosphorylation and fatty acid beta-oxidation (Schmitt and An, 2017). In addition to the compartments found ubiquitously in all mitochondria, the mammalian hepatocyte mitochondria contain three enzymes of the urea cycle, which detoxifies ammonia into urea (Brusilow and Horwich, 2001). Ammonia is a nitrogen waste product of protein catabolism and an extremely potent neurotoxin that can cause brain damage when its concentration in body fluids exceeds 50 μM (Brusilow and Horwich, 2001). Therefore, the primary function of the urea cycle in mammals is to protect the central nervous system from the toxic effects of ammonia (Brusilow and Horwich, 2001; Caldovic and Tuchman, 2003). Six enzymes, N-acetylglutamate synthase (NAGS, EC 2.3.1.1), carbamylphosphate synthetase 1 (CPS1, EC 6.3.4.16), ornithine transcarbamylase (OTC, EC 2.1.3.3), argininosuccinate synthase (ASS, EC 6.3.4.5), argininosuccinate lyase (ASL, EC 4.3.2.1), and arginase 1 (ARG1, EC 3.5.3.1) are required for the conversion of ammonia into urea (Brusilow and Horwich, 2001). NAGS, CPS1, and OTC are located in the mitochondria and ASS, ASL, and ARG1 are cytoplasmic enzymes. Additionally, two transporters, an ornithine/citrulline transporter (ORNT) and an aspartate/glutamate transporter, also known as citrin or ARALAR2, are also required for normal function of the urea cycle (Bradford and McGivan, 1980; Kobayashi et al., 1999; Brusilow and Horwich, 2001). Aside from liver, NAGS, CPS1, and OTC are also expressed in the intestinal mucosa where they catalyze formation of citrulline—a precursor for nitric oxide and arginine biosynthesis in mammals (Brusilow and Horwich, 2001).

NAGS catalyzes formation of N-acetylglutamate (NAG), which is an essential allosteric activator of CPS1 in mammals. The amino acid sequence of the mammalian NAGS consists of three regions with different degrees of conservation: the mitochondrial targeting signal (MTS), the variable segment (VS), and the conserved segment (Caldovic et al., 2002a). When expressed in cultured insect cells, the mouse NAGS pre-protein was imported into the mitochondria and processed at two sites. Removal of the MTS resulted in a mature NAGS (NAGS-M) while removal of the MTS and the variable segment resulted in conserved NAGS (NAGS-C) (Morizono et al., 2004). Recombinant NAGS-M and NAGS-C both catalyze the formation of NAG and are activated by arginine (Caldovic et al., 2006).

CPS1 catalyzes the formation of carbamyl phosphate from ammonia, ATP, and bicarbonate, while OTC catalyzes the production of citrulline from carbamyl phosphate and ornithine (Brusilow and Horwich, 2001). Citrulline is exported into cytoplasm via ORNT and converted into ornithine and urea by ASS, ASL, and ARG1. Ornithine re-enters the urea cycle upon import into mitochondria by ORNT and urea is excreted by the kidneys (Brusilow and Horwich, 2001). CPS1 is presumed to be the rate-limiting enzyme of ureagenesis because increased protein catabolism due to either high protein diet or breakdown of cellular proteins does not result in the accumulation of downstream urea cycle intermediates (Waterlow, 1999).

NAGS, CPS1, and OTC are considered to be soluble matrix proteins (Clarke, 1976; Raijman, 1976; Raijman and Jones, 1976; Shigesada and Tatibana, 1978; Bendayan and Shore, 1982; Sonoda and Tatibana, 1983; Hamano et al., 1988). However, existing evidence suggests that instead of being uniformly distributed in the mitochondrial matrix these enzymes interact with each other and the inner mitochondrial membrane (IMM). For example, subcellular fractionation of rat liver mitochondria revealed that CPS1 and OTC interact with the IMM (Powers-Lee et al., 1987); a finding complemented by electron microscopy data showing that OTC is also closely associated with the IMM (Yokota and Mori, 1986). Studies using isotopic tracers and isolated mitochondria show channeling of urea cycle intermediates from CPS1 to OTC (Cohen et al., 1992) and from ASS to ASL to arginase 1 (Cheung et al., 1989). Clinical studies in patients with urea cycle defects who receive a liver transplant show a continued need for supplementation with arginine (Tuchman, 1989). This observation supports the idea that urea cycle intermediates tightly channel between urea cycle enzymes, which causes arginine, an intermediate of the urea cycle and a protein building block, to not leave the transplanted liver and hence require continued supplementation.

The above properties of the urea cycle suggest that mitochondrial urea cycle enzymes interact, allowing compartmentalization of urea cycle in the mitochondria. However, structural details of the urea cycle enzymes that allow such interaction and its clinical impact remains poorly studied. Using a combination of protein structural analysis, mapping of patient mutations, liver mitochondrial fractionation, co-immunoprecipitation of urea cycle enzymes, and super-resolution microscopy we provide structural evidence that NAGS, CPS1, and OTC enzymes interact and form clusters in the mitochondria. This evidence offers direct support that the urea cycle is yet another mitochondrial function that relies upon compartmentalization by the formation of a protein cluster.

## Materials and Methods

### Ethics Statement

Experimental procedures involving animals were approved by the Institutional Animal Care and Use Committee of the Children’s National Medical Center.

### Determination of the Solvent Accessible Surface Area and Conservation

Crystal structures 5DOT, 5DOU, and 1OTH were used to calculate relative solvent accessible surface area (SASA) for the apo and liganded CPS1, and OTC trimer structures after removal of heteroatoms and water molecules. SASA of each amino acid was calculated with the Shrake and Rupley dot method (Shrake and Rupley, 1973) as described by Ho^1^ and using mesh density 9,600. A custom Python script^2^ was used to calculate SASA for each residue. The same method was used to calculate maximal SASA for amino acid using polypeptide in which each of the 20 amino acids is flanked by a glycine residue (Supplementary File S1); this polypeptide was modeled as β-strand using VEGA 3.1.1 (Pedretti et al., 2002). Relative SASA was calculated by dividing SASA of each amino acid with its maximal SASA.

Conservation of amino acids was determined from the alignment of either 233 homologs of human CPS1 (Supplementary File S2) or 270 homologs of human OTC (Supplementary File S3) from vertebrates and multicellular invertebrates. Protein sequences were collected from the NCBI non-redundant protein sequence database using Protein BLAST (Altschul et al., 1990, 1997), default parameters (word size 6, expected threshold 10, scoring matrix BLOSSUM62, gap existence 11, and gap extension 1) and sequences of human CPS1 and OTC as queries. Clustal Omega (Madeira et al., 2019) was used for multiple protein sequence alignment and WebLOGO3 (Crooks et al., 2004) was used for visualization of multiple sequence alignments. Conservation of surface residues that are mutated in patients with CPS1 and OTC deficiencies was determined as percent of either 233 CPS1 or 270 OTC homologs that have the same amino acid as human protein at that position.

### Accurate Modeling of the Impact of Mutations

The structural models of CPS1 and OTC used in the prediction of the effects of point mutations were obtained after several modeling steps. First we modeled the missing loops, side-chains and termini into the existing structures of CPS1 and OTC (PDB entries 5DOU and 1C9Y, respectively) using MODELLER version 9.23 (Eswar et al., 2006). Arginine 270 in the crystal structure of OTC was reverted to glutamine according the sequence reported in the UniProtKB database (entry P0048) (UniProt Consortium, 2019).

The prediction of changes in protein stability (the ΔΔ*G*) and structure resulting from single amino acid substitutions was performed with the ddg_monomer application, as implemented in Rosetta version 3.11, following the high-resolution protocol (Kellogg et al., 2011). The protocol generated 50 models for both the wild-type and the point mutant. The ΔΔ*G* of the mutation was calculated as the difference in Rosetta energies between the three highest scoring wild−type structures and the three top−scoring mutant structures. Input structures were pre-minimized to reduce steric clashes. Distance restraints between Cα pairs within 9 Å of each other were part of the optimization to prevent the backbone from moving too far from the starting conformation. The ideal value for the restraint was taken as the distance in the original structure and the standard deviation on the harmonic constraint was set to 0.5 Å. The score12 weight function (Rohl et al., 2004) was used in all calculations. The crystallographic threefold symmetry was explicitly imposed on all OTC models both in MODELLER and Rosetta. Mutant proteins with ΔΔG of 0–2 kcal/mol were considered to have similar stability as the wild-type while mutant proteins with ΔΔG < 0 kcal/mol were considered to be more stable than the wild type protein.

### Identification and Computational Analysis of VS Sequences

Protein sequences of vertebrate NAGS were collected using Blastp to query vertebrate proteins in either NCBI nr or UniProt databases with human and zebrafish NAGS (Caldovic et al., 2002a, 2014). Default parameters (word size 6, gap opening and extension penalties 11 and 1, respectively, and BLOSUM62 scoring matrix) were used for the search, which resulted in 90 mammalian NAGS sequences (Supplementary File S4) and 61 NAGS sequences from fish, amphibians and reptiles (Supplementary File S5). The most likely translation initiation site for each NAGS sequence was determined by inspection of protein alignments with the corresponding genomic sequences and with human and zebrafish NAGS, performed using ClustaW in MEGA7 (Kumar et al., 2018); amino acids encoded by predicted exons located upstream of the exon that corresponds to exon 1 in human and zebrafish *NAGS* genes were removed. The boundaries of the VS were defined as sequences between the mitochondrial protein peptidase (MPP) cleavage site and the beginning of sequence homology with vertebrate-like N-acetylglutamate synthase-kinase from *Xanthomonas campestris* (XcNAGS-K), which does not have MTS and VS (Qu et al., 2007). Sequence alignments with mouse NAGS, which has experimentally determined MPP cleavage site (Caldovic et al., 2010), as well as MitoPorotII (Claros and Vincens, 1996) and MitoFates (Fukasawa et al., 2015) were used for prediction of MPP cleavage sites in NAGS sequences. The C-termini of VS were determined by sequence alignments of NAGS sequences with XcNAGS-K using ClustalW in MEGA7. The lengths, proline content and sequence identities of VS were determined using MEGA7. WebLOGO3 (Crooks et al., 2004) was used to visualize VS sequence alignments that were generated with ClustalW in MEGA7.

### Fractionation of Rat Liver Mitochondria

Fractionation of mitochondria was carried out as described previously (Powers-Lee et al., 1987). Briefly, mitochondria were purified from donated rat livers using differential centrifugation (Graham, 2001). Purified mitochondria were resuspended in 5 mM Tris HCl, 250 mM Sucrose, 1 mM EDTA, pH 7.2, and subjected either to three rounds of freezing and thawing, or treatment with 0.12 mg of digitonin per mg of mitochondrial protein to remove the outer mitochondrial membrane as supernatant after centrifugation at 9000 × g for 10 min. Pelleted material was resuspended in 20 mM Hepes Buffer, pH 8.0 and sonicated. The vesicles that resulted from the sonication treatment were treated with increasing concentrations of Triton X-100 (0, 0.1, 0.5, and 1.0%) for 30 min. at room temperature, followed by pelleting of the membranes by ultracentrifugation at 144,000 × g for 60 min, washing three times with 20 mM Hepes, pH 8.0, and re-suspension in the same buffer. The amount of NAGS in each mitochondrial fraction was determined using immunoblotting with the primary antibody raised against recombinant mouse NAGS at 1:5,000 dilution and HPRT-conjugated donkey anti-rabbit secondary antibody (Pierce) at 1:50,000 dilution. NAGS bands were visualized using SuperSignal West Pico kit (Pierce) according to the manufacturer’s instructions. The amounts of CPS1 and OTC in each mitochondrial fraction were determined using immunoblotting with primary antibodies raised against CPS1 or OTC at 1:5,000 dilution, followed by the HPRT-conjugated secondary antibody at 1:10,000 dilution. CPS1 and OTC were visualized using ECL Western Blotting Substrate (Pierce) according to the manufacturer’s instructions. The intensity of each band was measured using a GS-800 Calibrated Densitometer (Bio-Rad) and the Quantity One software package (Bio-Rad). Mitochondrial fractions were probed with antibodies raised against mitochondrial markers of the IMM, mitochondrial matrix and outer mitochondrial membrane: CoxIV (Abcam) at 1:5,000 dilution, Grp75 (Stressgen) at 1:1,000 dilution and VDAC (Pierce) at 1:1,000 dilution (Da Cruz et al., 2003; Rardin et al., 2008, 2009). Filters were then probed with the HPRT-conjugated secondary antibody (Bio-Rad). The CoxIV, Grp75, and VDAC bands were visualized using ECL Western Blotting Substrate (Pierce).

### Cloning of Recombinant Mouse Variable Segments

Mouse variable segment (mVS) coding sequence was subcloned using pNS1 plasmid (Caldovic et al., 2002b) as a template and primers 5′-GGG ACA TAT GCT CAG CAC CGC CAG GGC TCA C-3′ and 5′-AGG TGG ATC CTT ATT ATT ACC AGT GGC GTG CTT CC-3′ which amplify the sequence between codons 49 and 117 of the mouse NAGS preprotein coding sequence. The amplification conditions were: initial denaturation at 95°C for 3 min., followed by 25 cycles of denaturation at 95°C for 30 s, annealing at 60°C for 30 s and extension at 72°C for 30 s, and final extension at 72°C for 5 min. using *Pfu* Turbo Hotstart DNA polymerase (Stratagene). This amplification product was cloned into pCR4Blunt-TOPO (Invitrogen) producing TOPOmVS. The correct coding sequence was confirmed by DNA sequencing. Plasmid TOPOmVS was cleaved with *Nde*I and *Bam*HI sites and subcloned into pET15b to create pET15bmVS.

The amino acid sequence of the reversed variable segment (revVS) was generated by reversing the amino acid sequence of mVS. The amino acid sequence of shuffled variable segment (shVS) was generated by dividing the sequence of mVS in the middle, then inter-digitating the amino acid sequences of the two halves. The coding sequences of revVS and shVS, including three stop codons at their 3′ ends and *Nde*I and *BamH*I restriction sites at the 5′- and 3′-ends, were chemically synthesized as mini-genes and inserted into pIDTSMART-KAN plasmid (Integrated DNA Technologies) followed by subcloning into pET15b bacterial expression vector to create pET15brevVS and pET15bshVS plasmids.

### Recombinant Protein Purification

Recombinant NAGS was purified as described previously (Caldovic et al., 2006; Haskins et al., 2008). Briefly, plasmid pET15bmNAGS-M (Caldovic et al., 2006) was used for overexpression of mouse NAGS-M in *E. coli*. Pelleted cells were resuspended in Buffer A (50 mM potassium phosphate, 500 mM KCl, 20% glycerol, 10 mM β-mercaptoethanol, 0.006%Triton X-100, 1% acetone, pH 7.5) containing 10 mM imidazole and lysed with 40 mM n-octyl-β-d-glucopyranoside. Cell lysate was loaded onto Ni-NTA agarose column and recombinant NAGS-M was eluted with Buffer A containing 250 mM imidazole.

Recombinant mVS, revVS, and shVS were purified from cultures of transformed *Escherichia coli* C41(DE3) cells that were induced with the Overnight Express Autoinduction Kit System 1 (Novagen). Cells were pelleted and resuspended in Buffer A containing 10 mM imidazole. Lysozyme and phenylmethylsulfonyl fluoride were added to the final concentrations of 1 mg/ml and 0.1 mM, respectively. The cells were lysed with 40 mM n-octyl-β-D-glucopyranoside. DNAseI and RNAseA (0.1–0.5 mg/ml lysate) in 5 mM MgCl_2_ were added to remove nucleic acids by incubation at room temperature for 30 min. Cell lysate was cleared by centrifugation at 25,000 × g for 30 min at 4°C. A nickel-affinity column (GE Healthcare) was equilibrated with buffer A containing 10 mM imidazole. Cleared lysate was loaded onto the column at a flow rate of 0.3 ml/min. The column was washed with Buffer A containing 50, 125, 250, and 500 mM imidazole. The variable segments eluted between 250 and 500 mM imidazole. The protein size and purity were verified by Comassie blue staining following SDS-PAGE on the 16.5% Tris-Tricine Gel (Bio-Rad).

### Mass Spectrometry Peptide Sequencing of Mouse Variable Segments

To confirm the identity of the purified mouse variable segments, they were excised from the 16.5% Tris-Tricine Gel and subjected to rapid, in-gel trypsin digestion (Shevchenko et al., 2006). The fragments were analyzed using mass spectrometry on an Applied Biosystems Voyager 4700 MALDI TOF/TOF mass spectrometer (Supplementary File S6).

### Co-immunoprecipitation

Mouse liver mitochondria were purified from donated tissue using differential centrifugation (Graham, 2001) and lysed with PBS containing 2% CHAPS (Stankiewicz et al., 2005). Mitochondrial lysate was diluted to 1 mg/ml protein for immunoprecipitation with antibodies against OTC and CPS1 and 5 mg/ml protein for immunoprecipitation with anti-NAGS antibodies. Mitochondrial lysates were mixed with magnetic beads (Invitrogen) cross-linked to primary antibodies against NAGS, CPS1 or OTC according to the manufacturer’s instructions. Following incubation at 20°C for 10 min, the beads were washed five times with PBS containing 0.05% Triton X-100. Protein complexes were eluted with ImmunoPure IgG Elution Buffer (Pierce). Protein concentration in each elution fraction was measured using protein assay dye reagent concentrate (Bio-Rad) according to the manufacturer’s instructions. Between 0.5 and 1 μg of immunoprecipitated proteins were resolved using SDS-PAGE, and probed with primary antibodies raised against NAGS, CPS1, or OTC followed by the HPRT-conjugated secondary antibody. NAGS was visualized using SuperSignal West Pico kit (Pierce), and CPS1 and OTC were visualized using ECL Western Blotting Substrate (Pierce).

In experiments measuring competition between NAGS-M and the recombinant mVS, mitochondrial lysate was diluted to a protein concentration of 2 mg/ml, mixed with the mVS, revVS, or shVS in a 1:1 (v/v) ratio, and added to magnetic beads (Invitrogen) cross-linked to primary antibodies against CPS1. Depending on the experiment, the molar excess of recombinant variable segment peptides relative to CPS1 was between 10 and 30-fold, based on estimates of the reported abundance of CPS1 in the liver mitochondria (Raijman, 1976; Cohen et al., 1982; Sonoda and Tatibana, 1983; Wang D. et al., 2019). Immunoprecipitation was carried out as described above. The intensities of NAGS-M bands were measured using a GS-800 Calibrated Densitometer and Quantity One software (Bio-Rad).

### Confocal and gSTED Microscopy

Confocal and Gated Stimulated Emission Depletion (gSTED) microscopy were performed as described previously (Bhuvanendran et al., 2014; Salka et al., 2017). Imaging was performed using the Leica TCS SP8 microscope equipped with a white light laser, two depletion lasers, acousto-optical beam splitter (AOBS) and hybrid detectors. Single labeling of all the confocal and gSTED samples was done using Alexa Fluor 647 while the double-labeled samples also had Alexa Fluor 532.

An HC PL APO CS2 100x/1.40 Oil objective was used to acquire confocal images. Alexa Fluor 532 was excited using 515 nm laser line and the emission was collected on a hybrid detector with the AOBS set to 520–590 nm whereas the Alexa Fluor 647 was excited using 645 nm laser line and the emission between 650 and 720 nm was collected.

12-bit gSTED images with pixel size less than 30 nm were acquired using the HC PL APO CS2 100x/1.40 Oil objective. Samples with Alexa Fluor 647 fluorophores were excited at 645 nm and depleted with 775 nm laser. The emission was collected between 650 and 720 nm with a time gating of 0.3–6.0 ns. In the double-labeled samples, sequential stack for Alexa Fluor 532 was acquired using a 515 nm excitation and 660 nm depletion. The time-gated emission between 2.2 and 6.0 ns was collected with the AOBS set from 520 to 590 nm.

These confocal and STED images were deconvolved with Huygens Professional version 17.04 (Scientific Volume Imaging^3^). Further image analysis, including intensity plots, were done using MetaMorph Premier version 7.7.0 (Molecular Devices^4^).

## Results and Discussion

### Comparative Analysis of CPS1 and OTC Surface Residues Whose Mutations Disrupt Ureagenesis and Cause Disease

More than half of CPS1 and OTC deficiency cases are caused by missense mutations (Haberle et al., 2011; Caldovic et al., 2015). Due to the tight channeling of CPS1 and OTC intermediates in the mitochondria (Cohen et al., 1992) we reasoned that some of the disease-causing missense variants of CPS1 and OTC might disrupt their interaction and used the following protocol to assess this possibility (Supplementary Figure S1). First, we compiled published reports of CPS1 and OTC missense mutations found in patients with clinical and biochemical symptoms of CPS1 and OTC deficiencies. Next, we calculated relative solvent accessible surface area (SASA) of CPS1 and OTC amino acids and mapped the positions of amino acids whose replacements cause CPS1 and OTC deficiencies. Mutations of surface amino acids, with relative SASA greater than 25% and known effects on the biochemical function of mutant CPS1 and OTC were not analyzed further. For mutations of surface residues whose effect on biochemical function of CPS1 and OTC has not been established, we calculated the effect of amino acid replacements on protein stability (Supplementary Figure S1). Similar analysis of NAGS missense variants could not be carried out as full-length NAGS crystal structure has not been determined. Only the acetyltransferase domain of human NAGS has been crystallized (Zhao et al., 2013), which is insufficient to build a homology model that accurately represents the NAGS quaternary structure.

**FIGURE 1:**
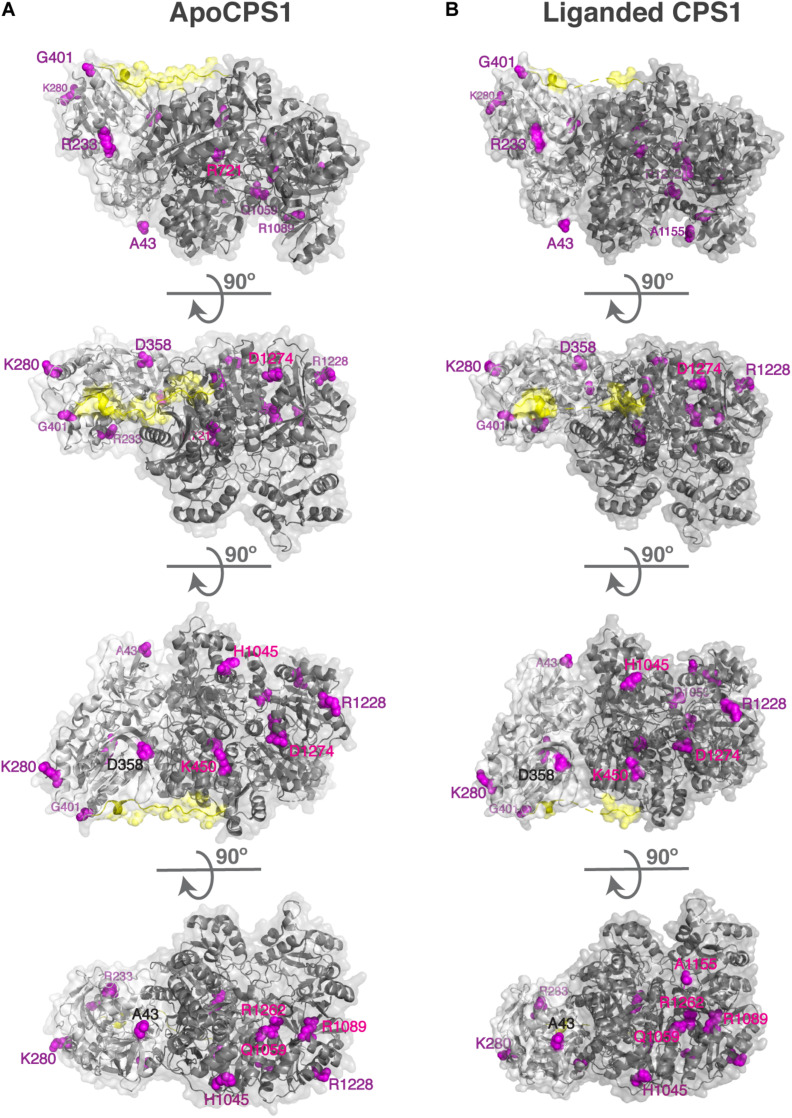
Surface residues in the apo **(A)** and liganded CPS1 **(B)** whose replacements cause CPS1 deficiency. Disease causing mutations in the *CPS1* gene were mapped to 3-dimensional structures of the apo CPS1 (5DOT) and liganded CPS1 (5DOU) enzymes. Surface residues in the apo and liganded CPS1 are shown in magenta. CPS1 glutaminase domain is shown in light gray, synthetase domain is shown in dark gray and the linker connecting the two domains is shown in yellow.

Human CPS1 pre-protein is 1,500 amino acids long; 1,352 and 1,422 amino acids are visible in the apo and liganded state, respectively (de Cima et al., 2015). Of these, 556 and 525 amino acids have more than 25% of their surface area exposed to solvent in the apo and liganded states, respectively, which we refer to as surface residues/amino acids (Levy, 2010). Of the 161 missense mutations that cause CPS1 deficiency (Supplementary Table S1), 22 mutations affect residues that are on the surface of both apo and liganded CPS1, 18 affect surface residues of the apo CPS1, and three affect surface residues of the liganded CPS1 (Supplementary Table S2). Biochemical properties of the p.Y389C, p.A438T, p.T471N, p.R721Q, p.K875E, p.E1255D, p.R1262Q, p.R1262P, p.C1327R, p.R1371L, p.P1439L, p.T1443A, and p.Y1491H recombinant CPS1 revealed that mutations of these surface residues result in destabilization, decreased enzymatic activity, and/or decreased affinity for NAG (Pekkala et al., 2009; Diez-Fernandez et al., 2013, 2014, 2015; Supplementary Table S2). The p.R1317W and p.G1333E replacements affect amino acids in the T’-loop and may disrupt substrate channeling between CPS1 active sites (de Cima et al., 2015), while replacement of the A438 with proline could affect flexibility of and hydrogen bonding within the T-loop (de Cima et al., 2015; Gao et al., 2015). To gain insight into effects of amino acid replacements on mutant CPS1 that were not characterized experimentally we modeled impact of 27 amino acid substitutions on CPS1 structure and calculated their effects on protein stability (Supplementary Table S2). Predicted stability of 15 mutant CPS1 proteins affecting 14 residues was either similar or higher than stability of the wild-type CPS1 (Supplementary Table S2). Replacements of these surface residues, distant to active and substrate binding sites, that do not destabilize the protein, qualify as mutations that could affect clustering of urea cycle enzymes by disrupting protein-protein interactions with OTC and/or NAGS (Figure 1 and Supplementary Table S2). Functional importance of the 14 surface amino acids whose replacements cause CPS1 deficiency was evaluated by determining their conservation in CPS1 homologs from 233 animal species (Supplementary File S2 and Supplementary Table S3). Eight of these residues are 100% conserved, and additional five are over 85% conserved in CPS1 homologs, while residues that correspond to less conserved H1045 and R1228 have similar size and/or chemical properties in most species (Table 1 and Supplementary Figure S2).

**TABLE 1:** Conservation of CPS1 surface residues whose replacements do not destabilize mutant protein and cause CPS1 deficiency.

Residue ID	Conservation score^a^	Amino acids in CPS1 and CPS3 from other species					
		Mammals	Birds	Reptiles	Amphibians	Fish	Invertebrates
A43	89	A	A/V/M	A	A	A/T/G	A/T/S/P
R233	100	R	R	R	R	R	R
K280	97	K	K	K	K	K/N/Q	K/Q/E/T
D358	100	D	D	D	D	D	D
G401	87	G/E/K/A/N/P	G/E/K	G/E	G	G/E/Q/W	G/K
K450	99	K/F	K	K	K	K	K
R721	100	R	R	R	R	R	R
H1045	42	H/Q	Q	Q	Q	H/Q/R/E	H/Q/E/S
Q1059	100	Q	Q	Q	Q	Q	Q
R1089	100	R	R	R	R	R	R
A1155	100	A	A	A	A	A	A
R1228	69 (90^b^)	R/W/Q	R/K/E	R/K	K	R/W/H/Q/C	R/K/Q/A
R1262	100	R	R	R	R	R	R
D1274	100	D	D	D	D	D	D

*^*a*^Conservation scores were determined as percent of 233 CPS1 and CPS3 homologs that have the same amino acid as human CPS1 at that position.*

*^*b*^Conservation of positively charged amino acids, R or K, in this position.*

**FIGURE 2:**
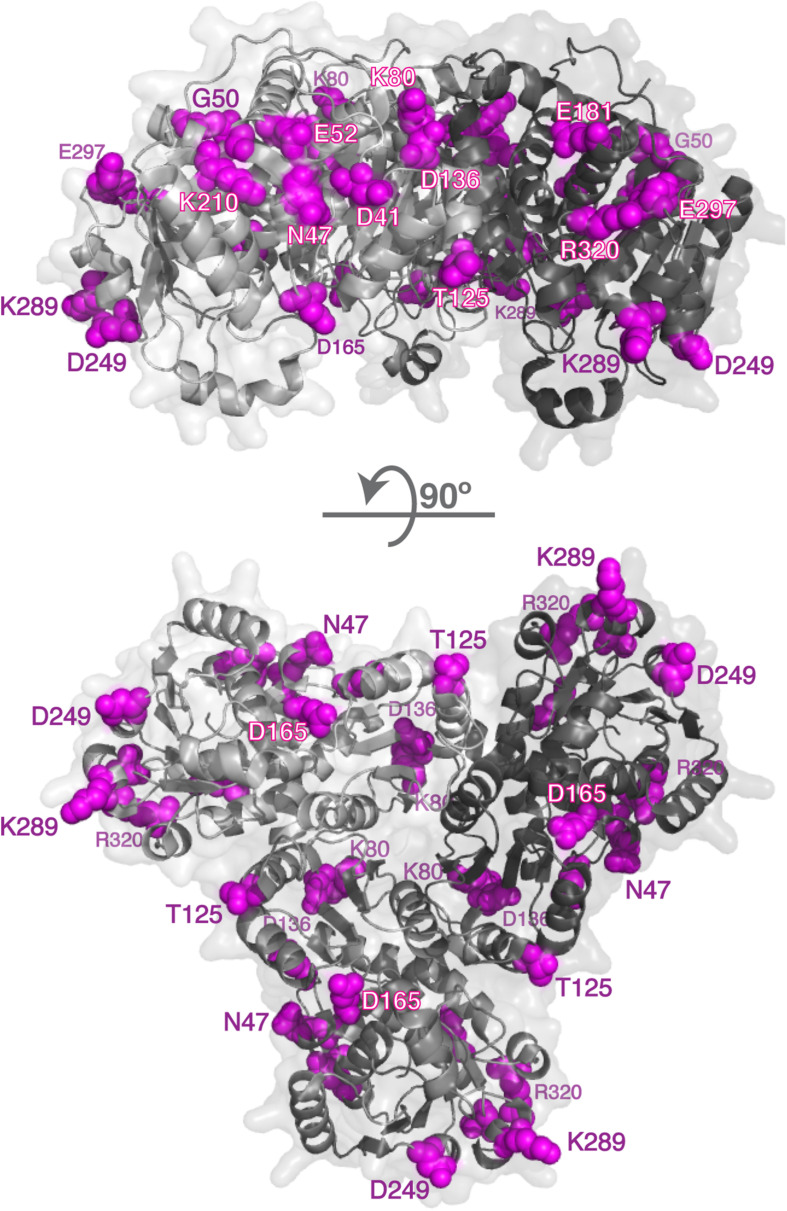
Surface residues in the OTC and whose replacements cause OTC deficiency. Disease causing mutations in the *OTC* gene were mapped to 3-dimensional structures of the human OTC (1OTH); 14 surface residues whose replacements cause OTC deficiency are shown in magenta. OTC monomers are shown in different shades of gray.

Mature human OTC protein is 322 amino acids long and the functional enzyme is a trimer (Shi et al., 1998; Caldovic et al., 2015). In each subunit 125 residues have over 25% of their surface area accessible to solvent. Of the 265 missense mutations that cause OTC deficiency (Supplementary Table S4), 51 are replacements of 35 surface amino acids. Deleterious effects of the p.R40H, p.T49P, p.A102P, p.H255P, p.Q270P, p.L349P, p.G269E, p.G269R, p.K221N, p.K289D, and p.K289N replacements can be explained by their experimentally tested effects on either *OTC* mRNA splicing or protein folding, stability, and catalytic properties of the mutant protein (Supplementary Table S5). Calculations of protein stability were used to predict effects of 41 amino acid substitutions affecting 27 surface amino acids on stability of mutant OTC; 18 mutant OTC were predicted to have similar stability as the wild-type protein (Figure 2 and Supplementary Table S5). As with CPS1, amino acid replacements of surface residues that are far from catalytic and substrate binding sites, and do not destabilize mutant protein could cause disease by disrupting interactions with CPS1 and/or NAGS. Functional importance of the 14 surface residues whose replacements do not destabilize mutant OTC was evaluated by determining their conservation in OTC proteins from 270 animal species (Supplementary File S3 and Supplementary Table S6). Of the 14 surface amino acids associated with deleterious missense mutations seven are conserved in 85% of OTC homologs and the remaining seven are replaced in most species by amino acids with similar size and/or chemical properties (Table 2 and Supplementary Figure S3).

**TABLE 2:** Conservation of OTC surface residues whose replacements do not destabilize mutant protein and cause OTC deficiency.

Residue ID	Conservation score^a^	Amino acids in OTC from other species					
		Mammals	Birds	Reptiles	Amphibians	Fish	Invertebrates
D41	66	D/H	D	D	D/N	H/S	D/H/N/T
N47	52	D/N/T	N	N	D/N/H	D/A	D/G/H/N/Y
G50	40	G/E/A	G/A/T/V	A/P	A	G/A/S/P	G/A/E/K/P/Q/R/S
E52	98	E/D	E	E/D	E/D	E	E/D
K80	99	K	K	K	K	K	K/R/H
T125	68	T/I/M/R/K	I/R	M/S	K/Q/R	T/A/G/V/K/R	I/L/K/R/N/Q/S/Y/P
D136	81	D	N	D	D	D	D
D165	91	D/E	D/N	D	D	D/E	D/E
E181	99	E	E	E	E	E	E/D
K210	97	K	K/Q	K/R	K	K	K/R/P
D249	91	D/E/N	D	D/N/V	D/E	D/E/N	D/N
K289	50	K	K/Q	E/Q	K/Q	K/E/Q/N	K/E/N
E297	72	D/N	N/D	D/G	D/N	D/N/G	D/E/N/H/R
R320	74	R/K/Q	R/Q	K/Q	R/K	R/H/L	R/K/H/N/Q/E

*^*a*^Conservation scores were determined as percent of 270 OTC homologs that have the same amino acid as human protein at that position.*

Conservation of surface amino acids in CPS1 and OTC that are mutated in patients with urea cycle deficiency suggests potential importance of these residues for the functioning of the urea cycle. It is possible that mutations of the surface residues that are predicted to have little effect on stability of the mutant proteins and are located far from the catalytic and substrate binding sites can cause disease by disrupting protein-protein interactions between CPS1 and OTC. Another possibility is that mutations of positively charged surface amino acids could disrupt interactions between CPS1, OTC and phospholipids such as cardiolipin that are enriched in the IMM. These two possibilities are not mutually exclusive and it is possible that some of mutations of surface amino acids primarily disrupt protein-protein interactions while others primarily disrupt protein-lipid interactions. Post-translational modifications of CPS1 and OTC lysine residues could disrupt their interactions either with each other or with phospholipids of the IMM. We note that CPS1 K280, and OTC K80 and K289, whose replacements could cause disease by disrupting the channeling of urea cycle intermediates, can be succinylated and/or glutarylated in human or mouse livers (Park et al., 2013; Tan et al., 2014). Removal of succinyl and glutaryl groups from lysine residues of CPS1 and OTC by SIRT5 (Du et al., 2011) could be a protective mechanism that ensures efficient ureagenesis, since SIRT5 deficient mice experience hyperammonemia (Nakagawa et al., 2009).

Recently, the three-dimensional structure 6UEL of human CPS1 liganded with an inhibitor intended for treatment of cancers became available (Yao et al., 2020). So, we used the 6UEL structure to repeat our analysis (Supplementary Figure S1). Of the 42 residues affected by mutations in patients with CPS1 deficiency and with relative SASA greater than 25% in 6UEL, 39 were identified as surface residues by our analysis of the 5DOT and 5DOU structures. The M729, S1203, and D1205 are mutated in patients with CPS1 deficiency, and their relative SASA is greater than 25% in the 6UEL structure. However, the biological significance of this result is unclear since 6UEL structure represents inactive form of CPS1 liganded with the H3B-193 inhibitor of CPS1 enzymatic activity (Yao et al., 2020). Therefore, solvent exposure of the M792, S1203, and D1205 residues in the 6UEL structure could well be a result of an inactive conformation induced by the CPS1 inhibitor.

### Protein-Protein Interactions Between NAGS, CPS1, and OTC in the Liver Mitochondria

**FIGURE 3:**
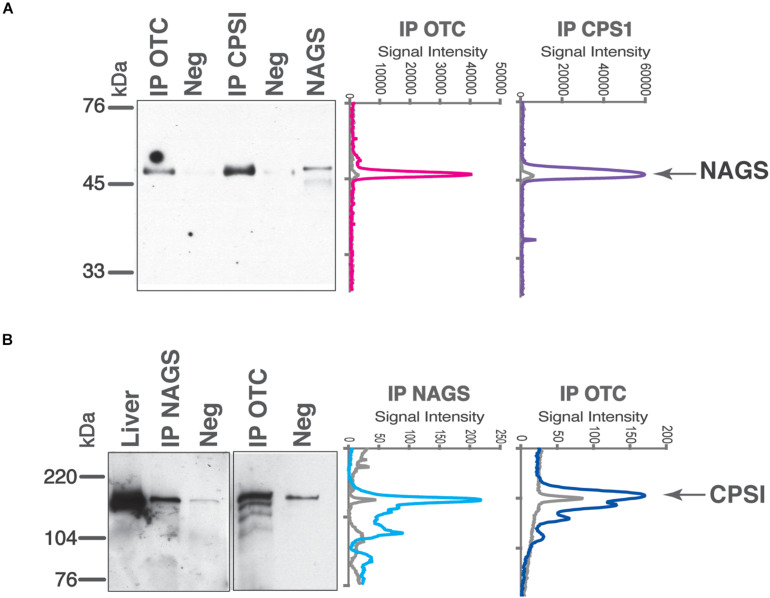
Co-immunoprecipitation of NAGS, CPSI, and OTC from the mouse liver. Anti-NAGS (IP NAGS lane), anti-CPSI (IP CPSI lane), and anti-OTC (IP OTC lane) antibodies were used for immunoprecipitation. Non-specific IgG (Neg) was used as a negative control. The precipitated proteins were probed with antibodies against NAGS **(A)** and CPSI **(B)**. Either 0.5 ng of recombinant NAGS-M and NAGS-C (panel A) or 5 μg (panel B) of liver proteins (Liver lane) were used as positive controls. Between 0.5 and 1 μg of immunoprecipitated proteins were resolved in IP NAGS, IP CPSI, and IP OTC lanes. Graphs on the right represent signal intensities of the bands representing co-immunoprecipitated proteins (colored lines) and their negative controls (gray).

While tight channeling of urea cycle intermediates (Tuchman, 1989; Cohen et al., 1992) suggests interactions between mitochondrial urea cycle proteins, large differences in the abundance of the three urea cycle enzymes in the mitochondria (Raijman, 1976; Cohen et al., 1982; Sonoda and Tatibana, 1983; Wang D. et al., 2019) would preclude formation of a NAGS-CPS1-OTC complex with a fixed stoichiometry. Protein clustering is a newly discovered phenomenon that can explain interactions among enzymes of highly disparate abundance that do not catalyze consecutive reactions of a metabolic pathway (An et al., 2008; Chan et al., 2015; French et al., 2016; Subramanian et al., 2019). With vastly different levels of NAGS, CPS1, and OTC proteins, we used co-immunoprecipitation to qualitatively examine if any interaction can be observed between NAGS, CPS1, and OTC. Proteins were immunoprecipitated using the protein-specific antibody, while non-specific antibody was used as a negative control. Purified recombinant NAGS (Figure 3A) and total liver proteins (Figure 3B) were used as positive controls for NAGS and CPS1, respectively. Anti-CPS1 antibody co-immunoprecipitated NAGS (Figure 3A), and conversely anti-NAGS antibody co-immunoprecipitated CPS1 (Figure 3B). Further, NAGS and CPS1 were both co-immunoprecipitated with the anti-OTC antibody (Figures 3A,B). While CPS1 co-immunoprecipitation showed partial non-specificity/degradation of CPS1, co-immunoprecipitation of the right sized band was significantly greater than the negative controls (Figure 3—line profiles). Co-immunoprecipitation of OTC, NAGS, and CPS1 enzymes with each other offered direct evidence in support of the ability these urea cycle enzymes to interact with each other in the liver mitochondria. With the presence of mitochondrial membrane vesicles in the lysates, these co-immunoprecipitations could also have been enhanced due to the interactions of surface residues of these proteins with the IMM.

**FIGURE 4:**
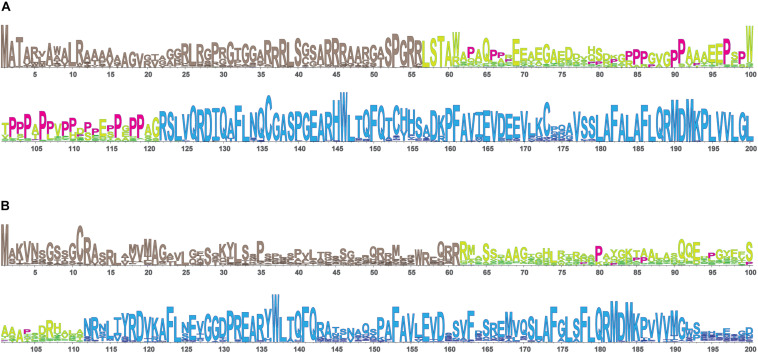
LOGO alignments of the N-terminal portions of NAGS from 90 mammals **(A)** and 61 fish, amphibians and reptiles **(B)**. NAGS is present in most vertebrates, but its efficient activity is crucial for survival of land dwelling organisms such as mammals that require highly efficient urea cycle. To examine protein sequence features associated with NAGS specialization in aquatic and land-dwelling vertebrates we compared alignments of NAGS from mammals and fish, amphibians and reptiles. There are three regions with differing degrees of conservation: N-terminal mitochondrial targeting signal, followed by VS, and conserved domain. Mammalian VS has higher proline content and greater sequence conservation than the VS from fish, amphibians and reptiles. Prolines are shown in magenta. Tan, mitochondrial targeting sequence; Yellow/lime green, variable segment (VS); Blue, conserved domain.

In view of the ability of the urea cycle proteins to interact with each other, we next examined the protein region responsible for promoting these interactions and focused on NAGS. We hypothesized that interactions between NAGS, CPS1, and OTC are crucial for increasing the efficiency of the urea cycle. While NAGS is present in all organisms, its efficient activity is crucial for land dwelling organisms such as mammals as their survival requires highly efficient disposal of nitrogenous waste (Cohen, 1963; Mommsen and Walsh, 1989; Haskins et al., 2008). Inversion of the allosteric effect of L-arginine from inhibition to activation of NAGS in land dwelling tetrapods is a feature of NAGS that enabled efficient ureagenesis (Haskins et al., 2008). Thus, we compared mammalian NAGS proteins with NAGS from fish, amphibians and reptiles; birds lack *NAGS* genes (Haskins et al., 2008). Variable segments (VS) from mammalian NAGS proteins are more conserved, longer and have higher proline content than VS from fish, amphibian and reptile NAGS proteins (Table 3, Figure 4, and Supplementary Figure S4). Because proline-rich protein segments can form extended, poly-proline type II helices that mediate protein-protein interactions (Rubin et al., 2000; Kelly et al., 2001; Ball et al., 2005) we used PPIIPred prediction software (O’Brien et al., 2020) to evaluate the ability of the VS in different taxonomic groups to form such a secondary structure. Formation of poly-proline type II helices by VS was predicted for mammalian NAGS (Supplementary Figure S5A), but not for fish, amphibian and reptile NAGS (Supplementary Figure S5B), suggesting interaction between mammalian NAGS and CPS1 may contribute to their efficient ureagenesis. In view of the co-immunoprecipitation of NAGS with CPS1 and predicted ability of mammalian VS to form secondary structure that can mediate protein-protein interactions, we examined if the VS mediates interaction of mammalian NAGS (Caldovic et al., 2002a) with CPS1 and whether this interaction may be important for NAGS function in the mammals.

**TABLE 3:** Properties of vertebrate VS.

	Mammals	Fish, Amphibians, and Reptiles
VS length	37–64	31–40
VS proline content	17.4–32.3%	2.3–11.1%
VS sequence identity	24–84%	34–60%

**FIGURE 5:**
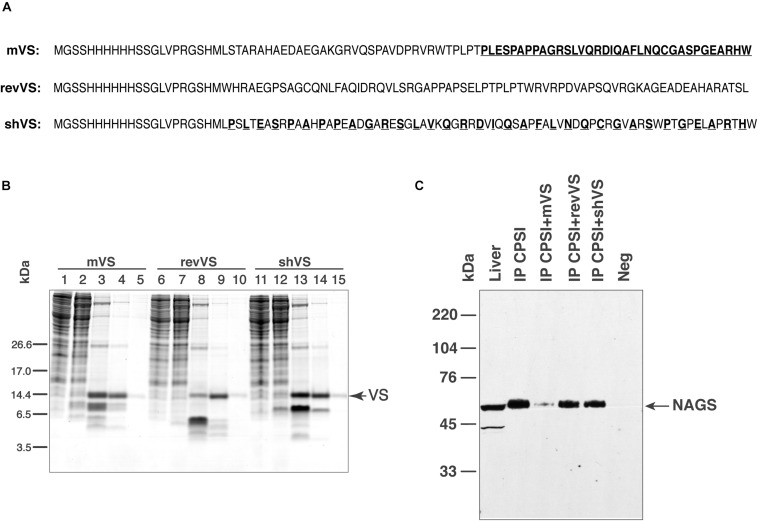
Competition between NAGS and mouse variable segment (mVS) for co-IP with CPSI. Recombinant mVS was overexpressed and purified from *E. coli*. **(A)** Amino acid sequences of recombinant mouse variable segment (mVS), reversed (revVS) and shuffled (shVS) variable segments. Amino acids from the C-terminal half of mVS (bold and underlined typeface) were interdigitated with amino acids from the N-terminal half to create shVS. **(B)** Purification of mVS, rVS and shVS. Lanes 1, 6 and 11—flow trough. Lanes 2, 7 and 12—wash with 50 mM imidazole. Lanes 3, 8 and 13—elution with 125 mM imidazole. Lanes 4, 9, and 14—elution with 250 mM imidazole. Lanes 5, 10 and 15—elution with 500 mM imidazole. **(C)** Anti-CPSI antibodies were used for immunoprecipitation of mitochondrial proteins. The precipitated proteins were probed with anti-mNAGS antibodies. Precipitation with non-specific antibodies (Neg), revVS (IP CPSI+revVS) and shVS (IP CPSI+shVS) were negative controls. Total liver proteins (Liver) were positive control.

To assess whether mammalian VS mediates NAGS-CPS1 interactions, we purified recombinant mouse variable segment (mVS) and examined its ability to compete with the endogenous NAGS for binding to CPS1. Two peptides with identical amino acid compositions as mVS, but with altered amino acid sequence: reverse mouse variable segment (revVS) and a shuffled mouse variable segment (shVS) polypeptide were controls for sequence specificity of the interaction (Figure 5A). Recombinant mVS, revVS, and shVS, tagged with poly-histidine at the N-terminus, were overexpressed in *E. coli* and purified using nickel affinity chromatography. Purified mVS, revVS, and shVS migrated as 9.5 kDa bands, which are in close agreement with their predicted molecular weight of 9.675 kDa (Figure 5B). Mass spectrometry peptide fingerprinting and peptide sequencing of purified mVS, revVS and shVS were used to confirm their sequences (Supplementary Figure S6 and Supplementary File S6). Recombinant mVS, revVS or shVS were added to liver mitochondrial lysate followed by co-immunoprecipitation using the anti-CPS1 antibody and probed with the anti-NAGS antibody. Total liver protein was used as positive control for immunoblotting. Separate co-immunoprecipitations were performed using non-specific IgG antibodies, revVS (IP CPS1+revVS) or shVS (IP CPS1+shVS). Addition of recombinant mVS, but not of revVS nor shVS, prevented mNAGS binding to CPS1, causing a fivefold decrease in the amount of mNAGS that co-immunoprecipitated with the CPS1 in the presence of mVS (Figure 5C).

Immunoprecipitation experiments suggest stable interaction between NAGS, CPS1, and OTC. The CPS1 monomer and OTC trimer, which are the active forms of these two enzymes, are present in approximately a 10:1 molar ratio in the liver mitochondria (Raijman, 1976; Cohen et al., 1982; Wang D. et al., 2019), while NAGS is approximately one thousand times less abundant than CPS1 (Sonoda and Tatibana, 1983; Wang D. et al., 2019). These differences in abundance of NAGS, CPS1, and OTC combined with their co-immunoprecipitation provide direct evidence in support of clustering of these urea cycle enzymes. It is possible that the NAGS is tethered to one molecule of CPS1 via VS and provides NAG to a number of neighboring CPS1 molecules. Another possibility is a dynamic interaction between NAGS, CPS1, and OTC may result in more than one complex of the three proteins. Clustering of NAGS, CPS1, and OTC during active catalysis of NAG, carbamylphosphate and citrulline formation could provide explanation for simultaneous stable interactions between three proteins and dynamic interaction between stoichiometrically disparate NAGS and CPS1 molecules.

### Distribution of NAGS, CPS1, and OTC in Liver Mitochondria

**FIGURE 6:**
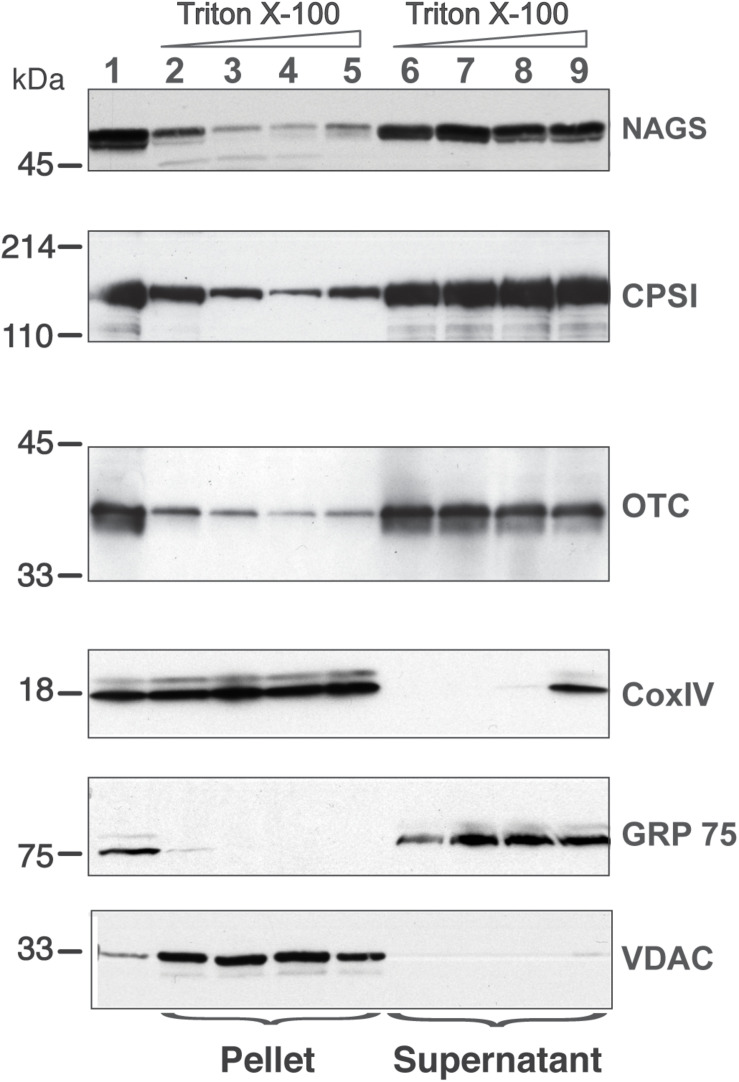
Distribution of NAGS, CPSI, and OTC in the liver mitochondria. Increasing amounts of TritonX-100 were added to mitochondrial membrane vesicles; proteins associated with the membrane (pellet) were separated from the soluble proteins (supernatant) and probed with the anti-NAGS, anti-CPSI, anti-OTC, anti-CoxIV, anti-Grp75, and anti-VDAC. 30 μg of liver mitochondrial proteins were used as positive control for NAGS and 2 μg of mitochondrial proteins was used as positive control for OTC, CPSI, COXIV, Grp75, and VDAC. Lane 1—liver mitochondrial proteins, lanes 2 and 6—0% TritonX-100, lanes 3 and 7—0.1% TritonX-100, lanes 4 and 8—0.5%, TitonX-100, lanes 5 and 9—1% TritonX-100.

While the above studies identify interaction between the urea cycle enzymes, they do not identify the location in the mitochondria where these interactions occur. Earlier fractionation studies found that CPS1 and OTC exist both in the mitochondrial matrix with a significant fraction loosely attached to the IMM (Powers-Lee et al., 1987). Thus, we used CPS1 and OTC as positive controls to examine NAGS distribution in the soluble and membrane-associated fractions of rat liver mitochondria. Glucose related protein 75 (Grp75), subunit IV of the cytochrome c oxidase (Cox IV), and voltage–dependent anion channel (VDAC) were used as markers of the soluble matrix, inner and outer mitochondrial membranes, respectively (Da Cruz et al., 2003; Rardin et al., 2008, 2009). These proteins confirmed the purity of the mitochondrial fractions (lanes 2 and 6 in Figure 6). The fraction of NAGS that partitioned with the membrane was 36 ± 19% (mean ± SEM, *n* = 3; lane 2 in Figure 6), while 63 ± 19% of NAGS was in the soluble fraction (lane 6 in Figure 6).

The addition of 0.1% Triton X-100 reduced the membrane-associated fraction of NAGS to 6 ± 2% (lane 3 in Figure 6). Increasing detergent concentrations, which dissociates more tightly bound proteins from membranes, did not increase solubilization of NAGS. Addition of Triton X-100 did not result in solubilization of Cox IV and VDAC (lanes 3–5 in Figure 6), while Grp75 remained soluble under the same experimental conditions (lanes 7–9 in Figure 6). Similar to our results with NAGS, and consistent with previous studies (Powers-Lee et al., 1987), 35 ± 5% of CPS1 and 30 ± 3% of OTC were associated with the IMM (Figure 6). Again, similar to NAGS, even at the highest detergent concentrations, CPS1 and OTC enzymes did not completely dissociate from the membranes. This suggests that part of the NAGS, OTC and CPS1 protein complex in the mitochondrial membrane exists in a detergent (0.1% Triton X-100)-resistant compartment. To further confirm that membrane association of these enzymes is not an artifact of the disruption of mitochondrial membranes by freezing and thawing, we carried out mitochondrial fractionation after solubilization of outer mitochondrial membrane with digitonin. This independently confirmed the distribution of NAGS, CPS1, and OTC at the IMM and in the matrix (Supplementary Figure S7).

**FIGURE 7:**
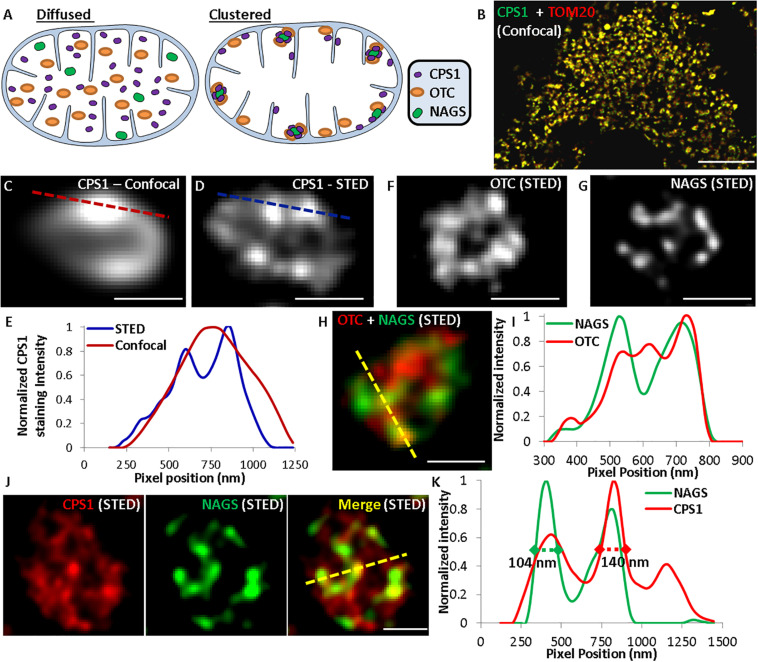
Use of super-resolution microscopy to determine *in situ* nanoscale organization of urea cycle enzymes. **(A)** Schematic representing the possible distribution of urea cycle enzymes in the mitochondria. **(B)** Confocal images showing localization of CPS1 and the mitochondrial protein TOM20 labeled with Alexa Fluor 532 and Alexa Fluor 647, respectively. **(C)** Confocal and **(D,F,G)** gSTED images showing individual mitochondria in mouse hepatocytes immuno-stained for **(C,D)** CPS1, **(F)** OTC, **(G)** NAGS, **(H)** OTC (Alexa Fluor 647) and NAGS (Alexa Fluor 532), **(J)** CPS1 (Alexa Fluor 647) and NAGS (Alexa Fluor 532). Single labeling in images **(C,D,F,G)** was done with Alexa Fluor 647. **(E,I,K)** Normalized pixel intensity plot for the dotted lines marked in the corresponding confocal or STED images.

The above biochemical studies established that of NAGS, CPS1, and OTC partition between IMM and mitochondrial matrix, which perhaps regulates the reserve capacity for ureagenesis in the matrix. These results suggest that of the two extreme cases outlined in Figure 7A—freely diffusing in the matrix (left) or clustered and bound to the IMM (right), the urea cycle enzymes may exist in the latter (clustered) state. To directly visualize the localization of the urea cycle enzymes *in situ* and assess if they are present in a diffused or clustered state, we used gated Stimulated Emission Depletion (gSTED) super-resolution microscopy to monitor nanoscale localization of NAGS, CPS1, and OTC in primary mouse hepatocytes. If these urea cycle enzymes are uniformly distributed in the mitochondrial matrix, then we would expect lack of any co-localized protein clusters (Figure 7A, left), whereas interactions of the three proteins with each other at the IMM would result in observation of NAGS, CPS1, and OTC enzyme clusters (Figure 7A, right). Confocal microscopy established the mitochondrial localization of the urea cycle enzymes (Figure 7B). Use of gSTED microscopy identified that, in addition to some diffuse localization in the mitochondrial matrix, these enzymes are all detectable in clusters away from the mitochondrial matrix. These clusters were 100–150 nm in size, and thus small enough to be below the resolution limit of confocal microscopy (Figures 7C–G). By co-immunostaining we observed that these clusters were not formed by individual proteins, but contained multiple urea cycle enzymes—NAGS and OTC (Figures 7H,I), and NAGS and CPS1 (Figures 7J,K). These *in situ* results support the interaction observed between NAGS, CPS1, and OTC by our biochemical co-localization studies. While some NAGS and OTC co-clustered, as indicated by overlapping fluorescence peaks for green (NAGS) and red (OTC) pixels (Figure 7I), we did observe OTC, NAGS, and CPS1 clusters that did not co-localize (Figures 7H–K). We observed that such independent clusters were greater for the abundant urea cycle enzyme, CPS1 (Figures 7J,K). Thus, the *in situ* super-resolution visualization approach identified that, while a proportion of the more abundant urea cycle enzymes (OTC, CPS1) co-localize with NAGS clusters, these proteins can also exist in independent clusters (red only peaks in Figures 7I,K), and can be detected more diffusely along the IMM or in the mitochondrial matrix (Figures 7H,J). NAGS, the less abundant and potentially regulatory urea cycle protein, was only detected in co-clusters with CPS1/OTC, as indicated by the spatial overlap of green (NAGS) with the red (CPS1/OTC) peaks in Figures 7I,K.

Our co-immunoprecipitation and super resolution microscopy data demonstrate that NAGS, CPS1 and OTC interact with each other and are present in a cluster along the IMM. While co-immunoprecipitation of the three proteins suggests stable interactions, the large difference in their abundance (Raijman, 1976; Cohen et al., 1982; Sonoda and Tatibana, 1983; Wang D. et al., 2019) raises questions regarding stoichiometry of such a complex. Our data, taken together with large differences in abundance of NAGS, CPS1, and OTC, are consistent with on-demand clustering of the three proteins at the IMM, similar to enzymes for biosynthesis of coenzyme Q and purines (An et al., 2008; Chan et al., 2015; French et al., 2016; Subramanian et al., 2019). Enzyme clustering provides metabolic advantages without the need for evolution of complimentary protein-protein interfaces (Sweetlove and Fernie, 2018). Theoretical modeling and experiments with engineered proteins in *E. coli* show that clustering of enzymes that catalyze consecutive reactions of a metabolic pathway can increase flux through the pathway by 100-fold; this is achieved through increased local concentrations of enzymes and metabolites in the cluster (Castellana et al., 2014). Another advantage of enzyme clusters is protection of unstable, highly reactive metabolites, such as carbamyl phosphate, from hydrolyzing or reacting with other metabolites or surface residues of proteins (Sweetlove and Fernie, 2018). The identity of surface amino acids could be important for their ability to cluster. We identified replacements of highly conserved CPS1 and OTC surface residues that may disrupt their on-demand clustering and effective catalysis of citrulline formation, which would result in accumulation of ammonia. This is similar to disease-causing mutations at the surface of adenylosuccinate lyase that disrupt formation of purinosome, a cluster of enzymes in *de novo* purine synthesis (Baresova et al., 2012).

The mitochondrial space appears to be organized into sub-compartments containing enzymes of different metabolic pathways. Enzymes in the Krebs’ cycle, electron transport, and fatty acid oxidation pathways form multiprotein complexes (Sumegi and Srere, 1984; Robinson and Srere, 1985; Robinson et al., 1987; Velot et al., 1997; Wu and Minteer, 2015; Bulutoglu et al., 2016; Liang et al., 2018; Wang Y. et al., 2019; Xia et al., 2019). Similarly, mitochondrial urea cycle enzymes appear to form clusters that can accommodate large differences in the abundance of NAGS, CPS1, and OTC. Biochemical characterization of the interactions between NAGS, CPS1, and OTC is needed to establish the driving force for their clustering. This driving force could be electrostatic, hydrophobic, or both and may require presence of the IMM phospholipids. Additional super resolution imaging will provide information about on-demand organization of the NAGS-CPS1-OTC cluster and determine its spatial relationship to other urea cycle enzymes and with other mitochondrial metabolic pathways. This is important because production of each urea molecule consumes three ATP molecules and efficient ureagenesis may rely on the proximity of CPS1 and protein complexes of the ATP producing pathways. An understanding of the driving force for clustering of NAGS, CPS1, and OTC will enable development of new treatments designed to promote and stabilize their interactions when one of the enzymes is defective.

## Data Availability Statement

All datasets generated for this study are included in the article and Supplementary Material.

## Ethics Statement

The animal study was reviewed and approved by the Institutional Animal Care and Use Committee of the Children’s National Medical Center.

## Author Contributions

NH: co-immunoprecipitation of NAGS, CPS1 and OTC, overexpression and purification of recombinant mVS, revVS and shVS, competition of mVS and NAGS for co-immunoprecipitation with CPS1, mitochondrial fractionation and immunoblotting, immunofluorescence of NAGS, CPS1, and OTC clusters. SB: immunofluorescence and image analysis of NAGS, CPS1, and OTC clusters. CA: designing and performing *in silico* calculations of protein stability for mutant CPS1 and OTC. AG, TK, KKo, JL, KKr, DS, NS, and EB: carrying out bioinformatic analysis of the NAGS variable segment ass ad a class project in a graduate-level course taught by LC. MT: design of biochemical studies and critical revisions of the manuscript. HM: calculation of the relative SASA for CPS1 and OTC amino acids. JJ: design of the immunofluorescence studies, data analysis, and writing and revision of the manuscript. LC: mapping of the CPS1 and OTC patient mutations, design of biochemical studies, and preparation and revision of the manuscript.

## Conflict of Interest

The authors declare that the research was conducted in the absence of any commercial or financial relationships that could be construed as a potential conflict of interest.

## Footnotes

1 http://boscoh.com/protein/calculating-the-solvent-accessible-surface-area-asa.html

2 https://github.com/morizono/pdbremix

3 http://svi.nl

4 www.metamorph.com
