# Supplementary material for: Mitochondrial Enzymes of the Urea Cycle Cluster at the Inner Mitochondrial Membrane
Source: Front Physiol. 2021 Jan 21;11:542950. doi: 10.3389/fphys.2020.542950 (PMC7860981; doi:10.3389/fphys.2020.542950)
Supplement: Supplementary file 1 [file Data_Sheet_1.zip › SupplementaryData_DataSheet/Supplementary_Figures_Revision.pdf]

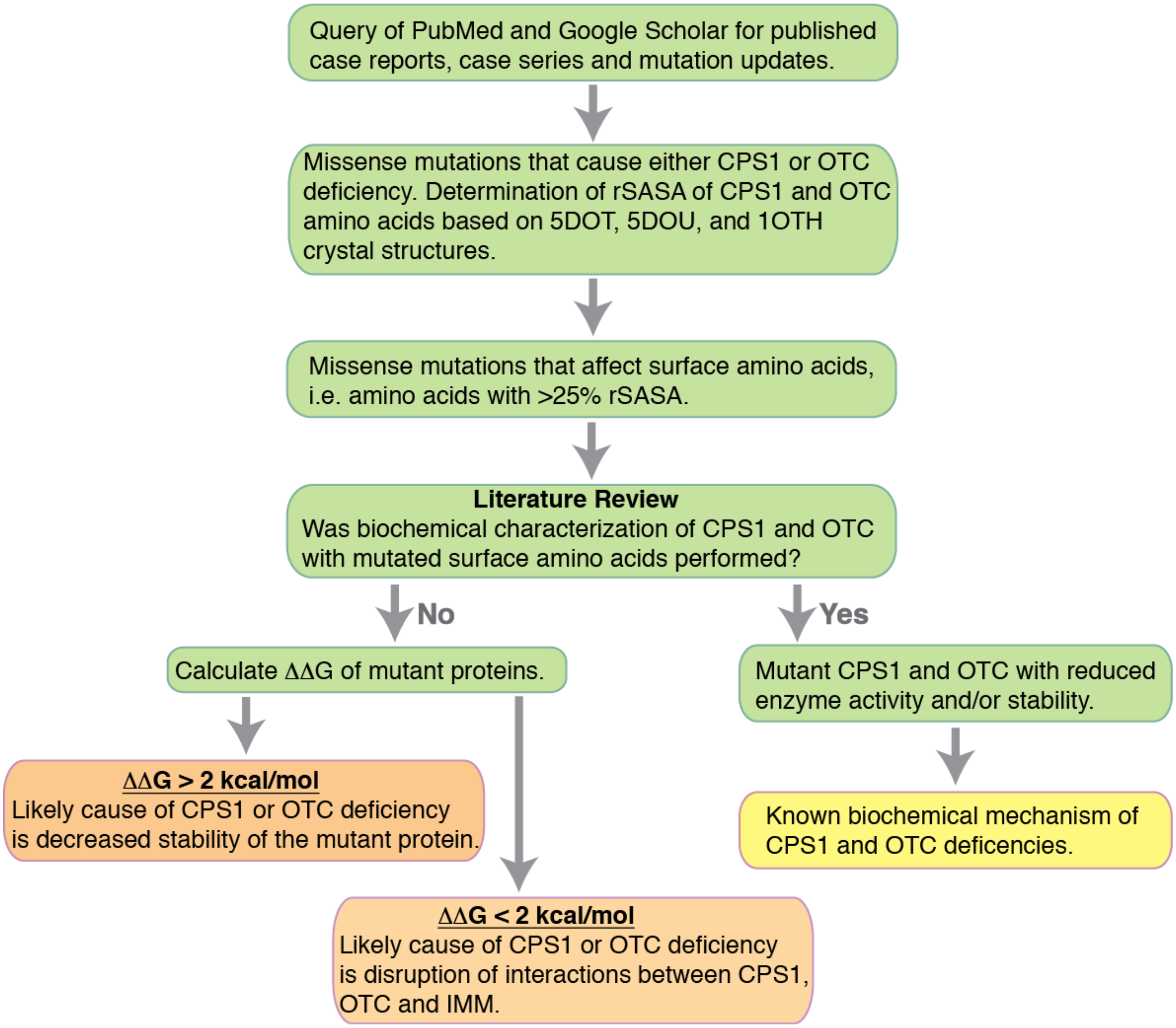

Figure S1. Workflow for evaluation of the biochemical mechanism by which CPS1 and OTC missense mutations cause CPS1 and OTC deficiencies.

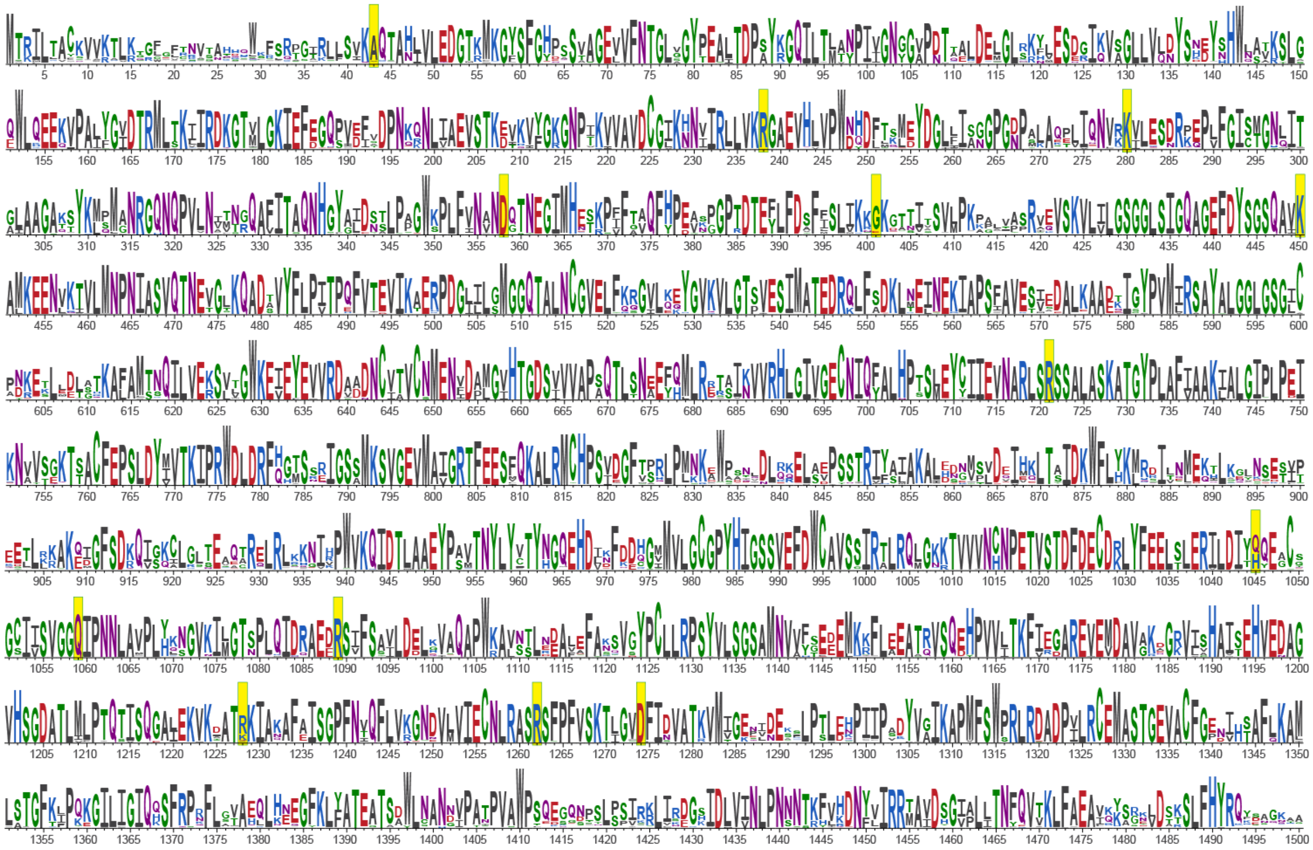

Figure S2. Conservation of 233 animal CPS1 and CPS3 protein sequences. The numbering corresponds to the human CPS1 sequence; amino acids are colored according to their chemical properties: blue – basic, res – acidic, purple – neutral, green – polar, black – hydrophobic. Letter size is proportional to amino acid conservation. Surface residues whose replacements do not destabilize mutant protein and cause CPS1 deficiency are highlighted in yellow.

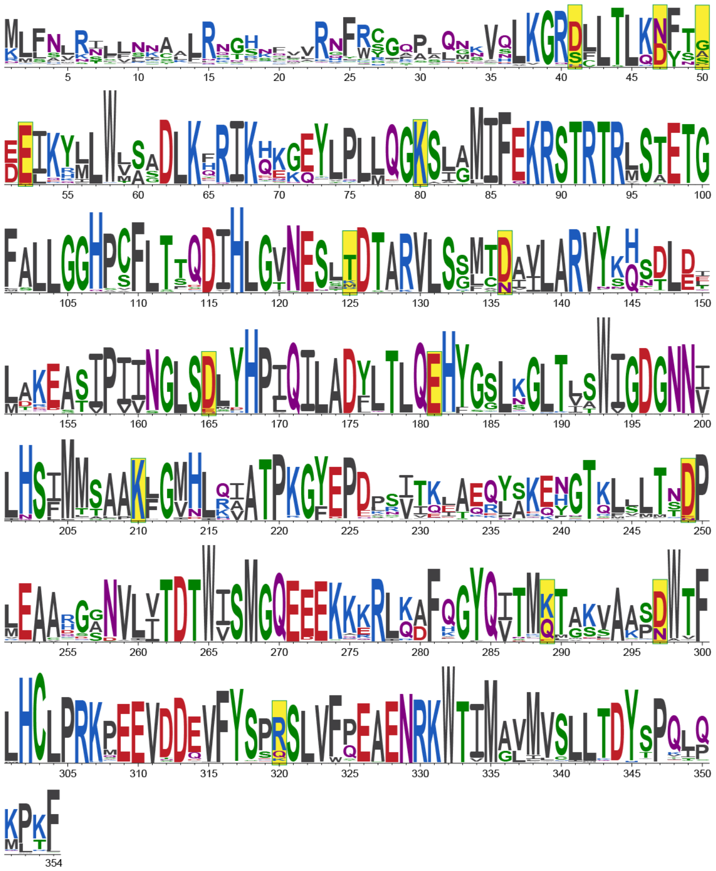

Figure S3. Conservation of 270 animal OTC protein sequences. The numbering corresponds to the human OTC sequence; amino acids are colored according to their chemical properties: blue – basic, res – acidic, purple – neutral, green – polar, black – hydrophobic. Letter size is proportional to amino acid conservation. Surface residues whose replacements do not destabilize mutant protein and cause OTC deficiency are highlighted in yellow.

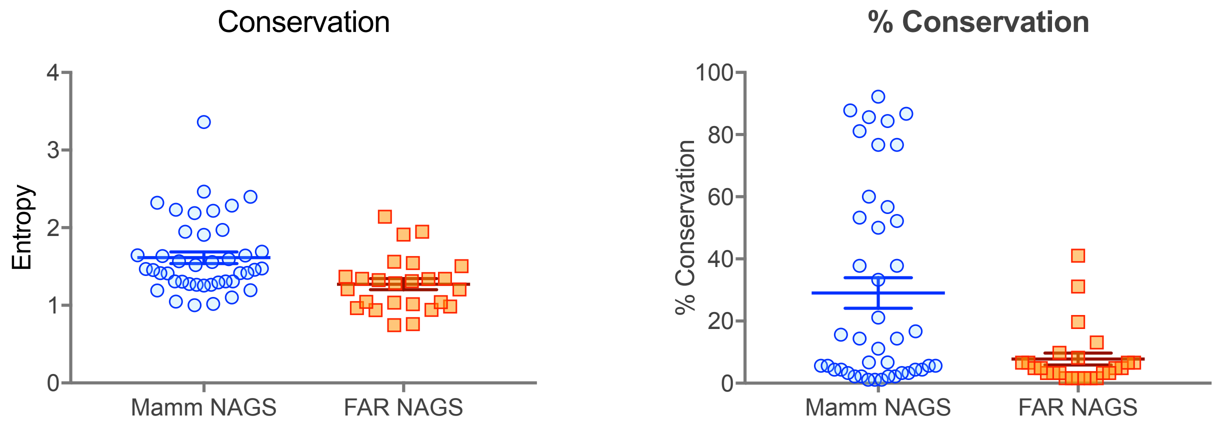

Figure S4. Overall conservation **(A)** and conservation of proline residues **(B)** in variable segments (VS) of mammalian NAGS (blue) and NAGS from fish, amphibians and reptiles (FAR, orange). **A.** Conservation of each VS residue was extracted from the raw data of the WebLogo alignments of mammalian NAGS and of FAR NAGS. WebLogo software assigns higher entropy values to more conserved residues in multiprotein alignments. **B.** Conservation of proline residues in the VS was calculated as percent of either mammalian or FAR NAGS that have a proline at each position of the multiprotein alignments.

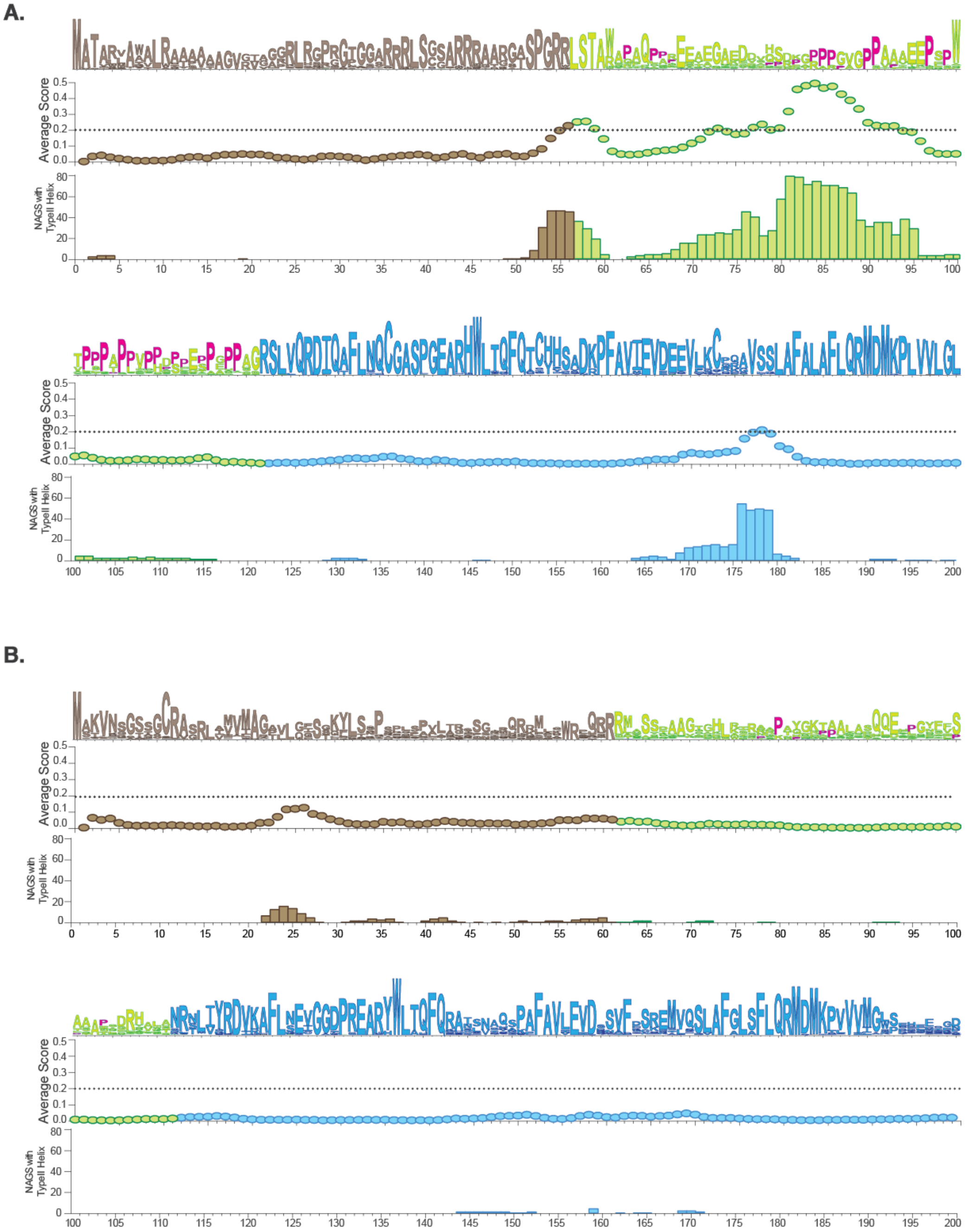

Figure S5. Predicted ability of VS to form poly-proline, type II helices in mammalian NAGS **(A)** and fish, amphibian and reptile NAGS **(B)**. Predictions are shown for the first 200 amino acids of the multiprotein alignments. The plot immediately below each Logo alignment show average scores for the probability of type II helix secondary structure in 90 mammalian NAGS (A) and 61 FARNAGS (B). Dotted lines indicate the threshold for predicted type II helical structure. The second plot below each Logo alignment shows the number of NAGS sequences with score for predicted type II helix above threshold at each amino acid position.

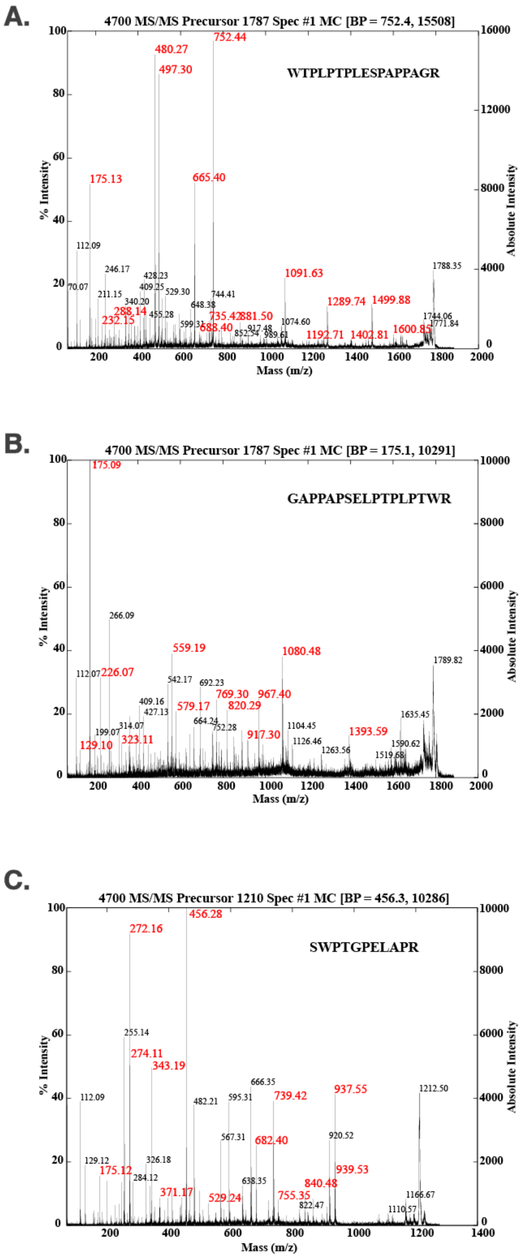

Figure S6. Mass spectra of purified mVS (A), revVS (B) and shVS (C).

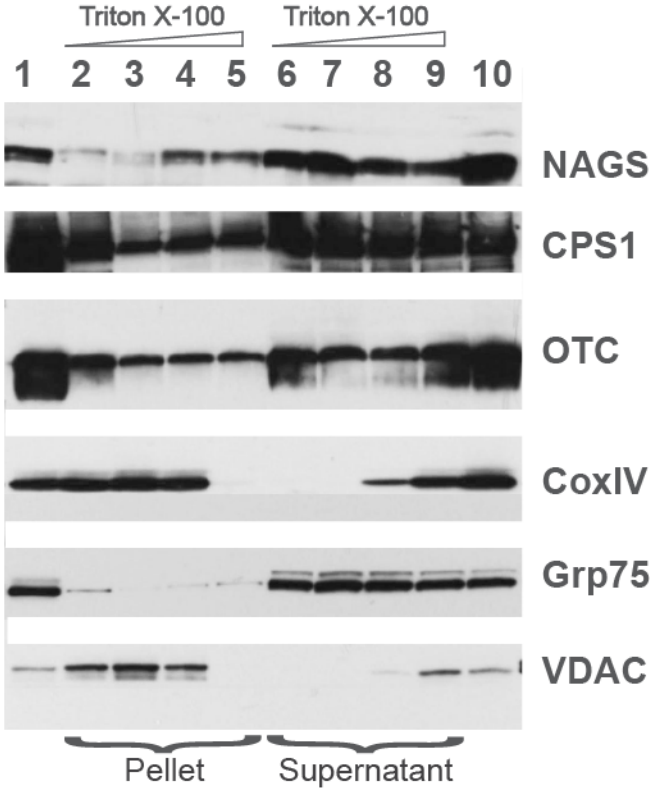

Figure S7. Distribution of NAGS, CPS1 and OTC in the liver mitochondria. Increasing amounts of TritonX-100 were added to sonicated mitoplasts after removal of OMM with digitonin; proteins associated with the membrane (pellet) were separated from the soluble proteins (supernatant) and probed with the anti-NAGS, anti-CPS1, anti-OTC, anti-CoxIV, anti-Grp75 and anti-VDAC antibodies. 30 µg of liver mitochondrial proteins were used as positive control for NAGS and 2 µg of mitochondrial proteins was used as positive controls for OTC, CPS1, COXIV, Grp75 and VDAC. Lane 1 – liver mitochondrial proteins, lanes 2 and 6 – 0% TritonX-100, lanes 3 and 7 – 0.1% TritonX-100, lanes 4 and 8 – 0.5% TitonX-100, lanes 5 and 9 – 1% TritonX-100, lane 10 – liver mitoplast proteins.
